# Breaking boundaries—coagulation and fibrinolysis at the neurovascular interface

**DOI:** 10.3389/fncel.2015.00354

**Published:** 2015-09-16

**Authors:** Sophia Bardehle, Victoria A. Rafalski, Katerina Akassoglou

**Affiliations:** ^1^Gladstone Institute of Neurological Disease, University of California, San FranciscoSan Francisco, CA, USA; ^2^Department of Neurology, University of California, San FranciscoSan Francisco, CA, USA

**Keywords:** fibrinogen, blood-brain barrier, microglia, autoimmunity, neuroinflammation, neurodegeneration, multiple sclerosis, Alzheimer’s disease

## Abstract

Blood proteins at the neurovascular unit (NVU) are emerging as important molecular determinants of communication between the brain and the immune system. Over the past two decades, roles for the plasminogen activation (PA)/plasmin system in fibrinolysis have been extended from peripheral dissolution of blood clots to the regulation of central nervous system (CNS) functions in physiology and disease. In this review, we discuss how fibrin and its proteolytic degradation affect neuroinflammatory, degenerative and repair processes. In particular, we focus on novel functions of fibrin—the final product of the coagulation cascade and the main substrate of plasmin—in the activation of immune responses and trafficking of immune cells into the brain. We also comment on the suitability of the coagulation and fibrinolytic systems as potential biomarkers and drug targets in diseases, such as multiple sclerosis (MS), Alzheimer’s disease (AD) and stroke. Studying coagulation and fibrinolysis as major molecular pathways that regulate cellular functions at the NVU has the potential to lead to the development of novel strategies for the detection and treatment of neurologic diseases.

## Fibrin Formation and Degradation in the CNS

The plasminogen activation (PA) system is an enzymatic cascade with key regulatory functions in fibrinolysis and degradation of extracellular matrix proteins (Syrovets and Simmet, 2004; Castellino and Ploplis, 2005; Kwaan, 2014). Plasminogen circulates in the blood as an inactive zymogen that is converted into active plasmin by tissue-type plasminogen activator (tPA) or urokinase-type plasminogen activator (uPA). The serine protease tPA is an immediate-early response gene expressed in the brain (Bignami et al., 1982; Qian et al., 1993; Sappino et al., 1993; Carroll et al., 1994; Tsirka et al., 1995). The activity of tPA is controlled by plasminogen activator inhibitor 1 (PAI-1). Upon activation, plasmin binds its main substrate fibrin(ogen) and degrades insoluble fibrin deposits that form intravascularly during blood clotting, as well as in the central nervous system (CNS) parenchyma after vascular rupture (Cesarman-Maus and Hajjar, 2005; Davalos et al., 2012). Fibrin controls plasmin activity through its capacity to bind plasminogen (Plg) as well as tPA or tPA/PAI-1 complexes to facilitate their proximate interaction (Wagner et al., 1989; Kaczmarek et al., 1993; Kim et al., 2012).

**Figure 1:**
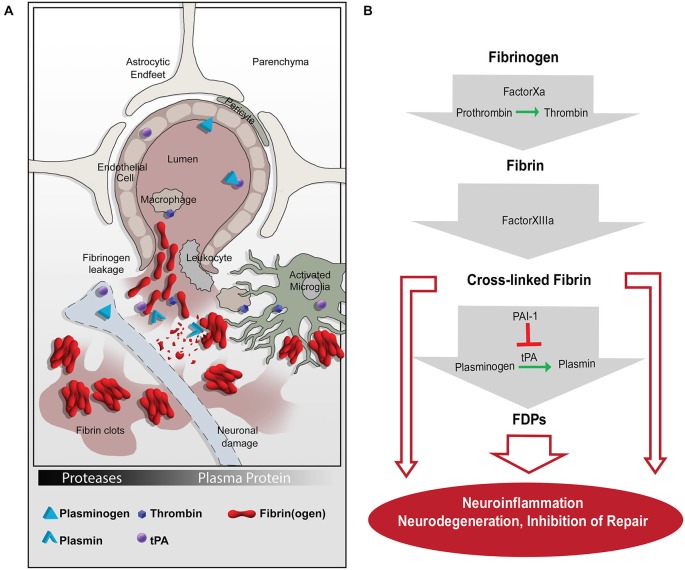
**The coagulation and proteolytic cascades at the neurovascular interface. (A)** Fibrinogen leakage in the central nervous system (CNS) and activation of the plasminogen activation (PA) system occur following blood-brain-barrier (BBB) disruption. The molecular network of fibrin and the PA system enable inflammation and neurodegeneration via activation of microglia, macrophages, and leukocytes. **(B)** A series of proteolytic events converts extravasated fibrinogen into insoluble fibrin, which can be cleaved into FDPs. Fibrin and FDPs interact with cellular receptors to induce inflammation, degeneration, and repair inhibition in the nervous system. tPA, tissue plasminogen activator; PAI-1, plasminogen activator inhibitor-1; FDPs, fibrin degradation products.

The pivotal fibrinolytic functions of the PA system were discovered in Plg-deficient mice, which show impaired wound healing, severe thrombosis, early lethality and delayed nerve regeneration (Bugge et al., 1995; Akassoglou et al., 2000). Interestingly, this phenotype is rescued by fibrinogen deficiency, suggesting that fibrin(ogen) is the main physiologic substrate for plasmin *in vivo* (Bugge et al., 1996; Akassoglou et al., 2000). Besides binding plasmin, fibrin(ogen) interacts with cell surface receptors expressed by different cell types in the CNS, including microglia (Adams et al., 2007; Davalos et al., 2012; Ryu et al., 2015), neurons (Schachtrup et al., 2007), astrocytes (Schachtrup et al., 2010) and Schwann cells (Akassoglou et al., 2002; reviewed in Davalos and Akassoglou, 2012; Ryu et al., 2009). Thus, fibrinogen acts as a molecular switch linking the PA system to activation of cell intrinsic signaling pathways involved in immune response and CNS homeostasis/neuronal functions (Figure 1).

The multifaceted and central functions of fibrin(ogen) in the PA system are highlighted by studies showing that fibrin acts: (1) as a main substrate of plasmin during fibrinolysis; (2) as a feed-back regulator of PA by binding tPA/PAI-1 or Plg directly; and (3) as a signaling molecule for cell activation in the CNS. By highlighting the PA system as a molecular link between coagulation, fibrinolysis and inflammation, this review will focus on cellular mechanisms and molecular signaling pathways driven by fibrin deposition and fibrinolysis in the CNS, specifically at the neurovascular unit (NVU).

## The Plasminogen System in Blood-Brain Barrier Dynamics

Under healthy conditions, plasma proteins like fibrinogen and Plg are not found in the brain parenchyma—a relatively immune-priviledged environment sealed by the selectively permeable blood-brain-barrier (BBB). Activation of the Plg system in the CNS parenchyma occurs in response to BBB disruption in which components from the blood enter the brain milieu (Figure 1). The BBB is an emergent property of the brain vasculature controlled by endothelial cells ensheathed by pericytes and astrocytic endfeet. The brain vasculature with an intact BBB plays essential roles in maintaining flow of nutrients into the brain, as well as protecting the brain from invasinto the brain, as well as protecting the brain from invasion by toxins, pathogens and inflammatory cells (Zlokovic, 2008; Daneman and Prat, 2015).

BBB opening can result from tight junction (TJ) complex disassembly or downregulation, increased transcellular transport, or physical damage to the blood vessel (Stamatovic et al., 2008). Disruption of the BBB is observed in a variety of neurological conditions in humans and in their animal models, such as stroke (Elster and Moody, 1990; Belayev et al., 1996), traumatic brain injury (Tanno et al., 1992; Conti et al., 2004; Shlosberg et al., 2010), epilepsy (Sokrab et al., 1990; Liu et al., 2012) and chronic neuroinflammation and neurodegeneration, including multiple sclerosis (MS; Paterson, 1976; Grossman et al., 1988; Miller et al., 1988; Adams et al., 2004; Gaitán et al., 2011) and Alzheimer’s disease (AD; van Oijen et al., 2005; Ahn et al., 2010; Cortes-Canteli et al., 2010; Oh et al., 2014b). BBB opening is also a hallmark of normal aging (Tucsek et al., 2014; Montagne et al., 2015). Indeed, contrast-enhanced MRI showed an age-dependent BBB breakdown in the hippocampus, a region critical for learning and memory that is affected in neurodegenerative diseases, such as AD (Montagne et al., 2015).

Multiple components of the PA system and in particular tPA function in BBB homeostasis (Vivien et al., 2011). tPA opens the BBB via mechanisms that include activation of platelet-derived growth factor-CC (PDGF-CC) signaling (Su et al., 2008), astrocyte remodeling through plasmin (Niego et al., 2012) and phosphorylation of BBB proteins claudin-5 and occludin (Kaur et al., 2011), as well as through a mechanism independent of its catalytic activity toward Plg (Abu Fanne et al., 2010). tPA may also open the BBB via low density lipoprotein receptor–related protein 1 (LRP-1) signaling (Yepes et al., 2003), which may be mediated by matrix metalloproteinase (MMPs; Wang et al., 2003; Lakhan et al., 2013). In contrast, PAI-1, the primary inhibitor of tPA, enhances barrier tightness in *in vitro* BBB models (Dohgu et al., 2011). tPA may also regulate the BBB through annexin-2 (Cristante et al., 2013). These studies show that tPA regulates several potentially overlapping pathways involved in BBB dysfunction. Evidence for tPA in maintaining vascular integrity can also be found in the clinic, as tPA treatment for thrombotic stroke increased hemorrhagic risk (Fugate and Rabinstein, 2014). Similarly, anticoagulants, such as clopidogrel, which inhibit platelet functions, increase the risk of brain hemorrhage after a stroke (Morrow et al., 2012).

In addition to the fibrinolytic system, molecular players promoting clot formation also regulate the BBB. Thrombin, the catalyst of fibrin formation, may disrupt the BBB (Lee et al., 1997; Liu et al., 2010) and in a human brain endothelial cell line can induce upregulation of intercellular adhesion molecule 1 (ICAM-1), Vascular cell adhesion molecule-1 (VCAM-1) and cytokines chemokine (C-C motif) ligand 2 (CCL2) and CX3CL1 (Alabanza and Bynoe, 2012). Fibrinogen increases endothelial cell permeability *in vitro*, in part by reducing expression of TJ proteins (Tyagi et al., 2008; Patibandla et al., 2009). The likelihood of BBB opening in response to fibrinogen may be increased under pathological conditions in which fibrinogen/fibrin accumulates on the blood vessel wall and in the parenchyma. A positive feedback loop whereby a precipitating event transiently opens the BBB, leading to the activation of the Plg and coagulation systems in the CNS, the components of which then further act to exacerbates BBB dysfunction can be envisaged. In sum, many pathologies are associated with BBB breakdown, indicated by persistent fibrin deposition inside the CNS. Therefore, fibrin has emerged as a potential target for development of diagnostic tools and therapeutic strategies (Conti et al., 2004; Adams et al., 2007; Craig-Schapiro et al., 2011; Ahn et al., 2014; Davalos et al., 2014).

## Plasminogen Activation and Fibrin Degradation in CNS Inflammation

Insofar as fibrin is necessary to stop hemorrhage, and plasmin can remove fibrin clots that block vital blood flow, the PA system has a beneficial role in the brain. However, dysregulation of the PA and coagulation systems are linked to inflammation, which is a common hallmark of many CNS pathologies, including the autoimmune disease MS (East et al., 2005; Marik et al., 2007; Han et al., 2008), as well as other chronic neuroimmune and neurodegenerative disorders (van Oijen et al., 2005; Paul et al., 2007).

MS is an autoimmune disease in which the myelin-producing oligodendrocytes are targeted for destruction by the immune system. Histopathology of human brain tissue shows focal fibrin deposition in MS plaques, indicative of perivascular inflammation and BBB disruption (Gay and Esiri, 1991; Kirk et al., 2003; Vos et al., 2005; Marik et al., 2007) that is also observed in MS mouse models (Paterson et al., 1987; Adams et al., 2004, 2007). Proteomic analysis of chronic active plaques from MS patients revealed a set of coagulation proteins uniquely present in active plaques, suggesting a role for the coagulation cascade in the development of MS pathology (Han et al., 2008). Indeed, MS lesions have increased levels of PAI-1 and less fibrin degradation and, thus, more sustained fibrin deposition than normal control tissue (Gveric et al., 2003). Fibrin depletion provides protection in a wide range of MS mouse models (Paterson, 1976; Akassoglou et al., 2004; Adams et al., 2007; Yang et al., 2011; Davalos et al., 2012). Studies of other Plg cascade components also support the hypothesis that fibrin deposition is a major instigator of experimental autoimmune encephalomyelitis (EAE). *tPA*^−/−^ mice have increased disease severity in EAE, which may be due to accumulated fibrin deposits and/or loss of fibrin-independent tPA functions in the CNS (Lu et al., 2002; East et al., 2005). Exacerbation of demyelination in *tPA*^−/−^ or *plg*^−/−^ mice after peripheral nerve injury is fibrin-dependent, since fibrin depletion rescues the damaging effects of tPA or Plg deficiency (Akassoglou et al., 2000). Furthermore, *PAI-1*^−/−^ mice have reduced EAE severity associated with increased fibrinolysis (East et al., 2008). It is important to underscore that fibrin and the tPA/plasmin system act in concert to exert the full effect of vascular-driven neuroinflammation. For example, inflammation and fibrin-induced neurodegeneration are reduced in *plg*^−/−^ mice, suggesting that multiple molecular players from the coagulation and fibrinolytic systems are needed for a full inflammatory and degenerative response (Hultman et al., 2014).

Emerging evidence suggests a pivotal role of fibrin in the regulation of CNS innate and adaptive immune responses (Davalos et al., 2012; Ryu et al., 2015; Table 1). Fibrin(ogen) interactions with microglia, macrophages, and neutrophils via integrin receptor CD11b/CD18 (also known as Mac-1, Complement Receptor 3 or integrin α_M_β_2_) were identified as direct activation pathways of innate immune response (Davalos and Akassoglou, 2012). Extravascular fibrin deposition stimulates recruitment and perivascular clustering of microglia in EAE lesions (Davalos et al., 2012), while deletion of fibrin or blockade of fibrin signaling protects from microglial activation and axonal damage in EAE (Akassoglou et al., 2004; Adams et al., 2007). A recombinant mutant thrombin analog similarly ameliorated EAE progression, corroborating the regulatory functions of thrombin-mediated fibrinogen/fibrin conversion during neuroinflammation (Verbout et al., 2015). Fibrin-induced activation of microglia via CD11b/CD18 induced secretion of cytokines and chemokines that stimulate recruitment of peripheral monocytes/macrophages (Ryu et al., 2015). Importantly, fibrin in the CNS white matter was sufficient to induce the infiltration and activation of myelin-specific T cells, suggesting a fibrin-induced innate immune-mediated pathway that triggers CNS autoimmunity (Ryu et al., 2015). Potential direct effects of fibrin on T cells might also play a role in autoimmune responses (Takada et al., 2010). Moreover, PA-mediated opening of the BBB and extracellular proteolysis facilitates T-cell extravasation and migration (Cuzner and Opdenakker, 1999; Yepes et al., 2003). Genetic and pharmacologic evidence point to CD11b/CD18 as the major receptor mediating the *in vivo* proinflammatory effects of fibrin in the CNS (Adams et al., 2007; Davalos et al., 2012; Ryu et al., 2015). In addition to CD11b/CD18, *in vitro* evidence indicates a role for toll-like receptor 4 (TLR4) in fibrin-induced macrophage activation (Smiley et al., 2001). Moreover, *in vitro* evidence suggests a role for fibrinogen in neutrophil activation (Skogen et al., 1988; Rubel et al., 2001). The relative contributions of these proinflammatory pathways in the CNS *in vivo* remain to be determined. Overall, fibrin(ogen) and tPA/plasmin can be potent modulators of neuroinflammation.

**Table 1:** **Fibrin(ogen) cellular targets at the NVU in neurologic diseases**.

Target	Functions	Receptors/Signaling pathways	Model	Reference
**Resident cells**
Microglia	Activation	CD11b/CD18	*In vitro*: microglia cultures	Adams et al. (2007)
	– Phagocytosis	RhoA, Akt, PI3K		Davalos et al. (2012)
	– Perivascular clustering		*In vivo*: EAE; FIE, AD animal models	Ryu et al. (2015)
	– Chemokine and proinflammatory gene expression			Paul et al. (2007)
	– ROS release
Astrocytes	Gliosis	TGFβ, Smad2, CSPGs	*In vivo*: stab wound injury; cortical fibrinogen injection	Schachtrup et al. (2010)
	– Scar formation	CSPGs
Neurons	– Axonal damage	β3-integrin, EGFR	*In vitro*: neuronal cultures	Schachtrup et al. (2007)
	– Inhibition of neurite outgrowth			Davalos et al. (2012)
			*In vivo*: EAE, spinal cord injury, ischemic stroke	Ill-Raga et al. (2015)
Endothelial cells	– Increased permeability	ICAM-1, α5β1 F-actin, TJ proteins, MEK, ERK, VE-cadherin, fibrin fragment E and Bβ15–42, RhoGTPase	*In vitro*: endothelial cell cultures	Tyagi et al. (2008)
	– Infiltration of leukocytes			Patibandla et al. (2009)
				Jennewein et al. (2011)
				Muradashvili et al. (2011)
**CNS infiltrating cells**
T cells	– Recruitment	APC CD11b/CD18	*In vitro*: T cell/APC co-cultures	Ryu et al. (2015)
	– Activation	CXCL10, IL12, IFN-γ
	– Proliferation		*In vivo*: FIE; 2D2 TCR MOG transgenic mice	
	– Th1 differentiation
Macrophages	– Recruitment	CD11b/CD18	*In vitro*: macrophage cultures	Ryu et al. (2015)
	– Chemokine expression	TLR4		Smiley et al. (2001)
	– Infiltration	CXCL10, CCL2, MCP-1	*In vivo*: FIE

## Plasminogen Activation and Fibrin Degradation in Neurodegeneration and Repair

The PA system plays a critical role in normal cognitive function (e.g., regulation of synaptic plasticity) and neural dysfunction (Melchor and Strickland, 2005). For example, tPA can modulate neurotoxicity as *tPA*^−/−^ mice exhibit less neuronal death after hippocampal kainate injection or after ethanol withdrawal, both of which induce neurodegeneration (Tsirka et al., 1995; Skrzypiec et al., 2009). Unlike tPA and plasmin, fibrinogen is not present in the healthy brain. However, fibrinogen is detected in the brains of patients with MS (Gay and Esiri, 1991; Kirk et al., 2003; Vos et al., 2005; Marik et al., 2007), schizophrenia (Körschenhausen et al., 1996), HIV-encephalopathy (Dallasta et al., 1999), ischemia (Massberg et al., 1999), AD (Paul et al., 2007; Ryu and McLarnon, 2009) and normal aging (Viggars et al., 2011), all conditions which have transient or long-lasting BBB opening.

AD is a common aging-related neurodegenerative disease of dementia and is characterized by extracellular aggregates of beta-amyloid (Aβ) plaques and intracellular neurofibrillary tangles of tau protein (Huang and Mucke, 2012). Co-localization of microhemorrhages and amyloid plaques in human AD brains suggests that bleeding can precipitate or promote plaque deposition (Cullen et al., 2006). Fibrin deposits colocalize with areas of neurite dystrophy in human AD tissue and AD mouse models (Cortes-Canteli et al., 2015). Individuals with high levels of plasma fibrinogen have an increased risk for developing AD and dementia (van Oijen et al., 2005; Xu et al., 2008). Furthermore, AD patients with two alleles of *apoE ε4*, which is the strongest genetic risk factor for AD (Mahley and Huang, 2012), have significantly more fibrin deposition than AD patients with ε2 or ε3 apoE alleles (Hultman et al., 2013). Fibrin depletion in AD model mice via genetic and pharmacological methods ameliorates the disease pathology and cognitive impairment (Paul et al., 2007; Cortes-Canteli et al., 2010, 2015). AD model mice lacking one allele for *tPA* develop more severe Aβ plaque deposition and cognitive impairment (Oh et al., 2014a). This effect may be due to reduced fibrinolysis, but there is also evidence that tPA is neuroprotective via a fibrin-independent mechanism by promoting Aβ degradation (Melchor et al., 2003), perhaps by activating microglia to phagocytose Aβ plaques. The physical association of fibrin and Aβ impairs fibrin degradation, which has the potential to induce chronic inflammation (Ahn et al., 2010; Cortes-Canteli et al., 2010; Zamolodchikov and Strickland, 2012). This interaction seems to be instrumental in the disease process as administration of a peptide that inhibits fibrin-Aβ interaction rescues cognitive decline in AD mice (Ahn et al., 2014). An important question to address is whether Aβ plaques associated with fibrin exacerbate neurodegeneration.

Studies indicate that fibrinogen and the PA system also impacts nervous system repair through regulation of neuron-glia interactions. Regeneration in the CNS may be limited by the development of astrogliosis via fibrin-induced transforming growth factor beta (TGF-β) signaling in astrocytes (Schachtrup et al., 2010) or by fibrinogen-mediated inhibition of neurite outgrowth (Schachtrup et al., 2007; Table 1). In the peripheral nervous system, fibrin impedes remyelination by inhibiting Schwann cell migration and differentiation into myelinating cells (Akassoglou et al., 2002, 2003). The increased severity of nerve injury in *tPA*^−/−^ or *plg*^−/−^ knock-out mice in the sciatic nerve crush model is rescued by genetic or pharmacological fibrinogen depletion (Akassoglou et al., 2000; Siconolfi and Seeds, 2001), supporting the concept that fibrin accumulation is an important trigger for inhibition of remyelination. While these findings are highly suggestive of new pathways for fibrin and tPA/plasmin in regeneration, more work will be needed to determine their contribution as inhibitors of nervous system repair.

## Future Directions

Emerging evidence from the fields of neuroscience, immunology, and vascular biology have aimed the spotlight on fibrin and the fibrinolytic system for their pleiotropic functions in neurological diseases. Although current evidence points to fibrin as a major contributor to neuroinflammation and neurodegeneration, it is possible that other components of the coagulation cascade are activated upon neurologic disease and play a role in CNS diseases via fibrin-dependent and potentially fibrin-independent mechanisms (Akassoglou, 2015). For example, a novel molecular probe for thrombin identified increased thrombin activity in animal models of stroke (Chen et al., 2012) and MS (Davalos et al., 2014). In accordance, depletion of thrombin by anti-coagulants inhibits fibrin formation and is protective in MS animal models (Adams et al., 2007; Han et al., 2008; Davalos et al., 2012). It is now timely for the fields of neuroscience and neurology to explore the contribution of the coagulation cascade in inflammatory, degenerative, and repair processes in the CNS.

Fibrin degradation products (FDPs) are commonly used as biomarkers to assess the severity of trauma after injury, in sepsis, or myocardial infract. Components of the coagulation cascade and FDPs have been detected in MS patients (Aksungar et al., 2008; Han et al., 2008; Liguori et al., 2014), in patients with mild cognitive impairment (Xu et al., 2008), and in human AD (Cortes-Canteli et al., 2015; Zamolodchikov et al., 2015). However, most of these studies have been performed in small population cohorts without availability of imaging data, response to treatments, and disease duration. Studies in large patient cohorts would be required to assess whether components of coagulation or the fibrinolytic cascade correlate with disease progression in neurologic diseases. Although coagulation and fibrinolysis could trigger and perpetuate neurologic disease, animal models of vascular-driven inflammation and neurodegeneration are currently lacking. Inducing neuroinflammation in the CNS in Fibrinogen-induced encephalomyelitis (FIE) by introducing fibrinogen in the brain (Ryu et al., 2015), or perhaps by manipulating PA, or by transgenic or pharmacological models that increase BBB permeability could lead to vascular-driven experimental settings to study disease pathogenesis in the CNS.

Several FDA-approved drugs target different aspects of the coagulation cascade leading to reduced fibrin formation. Although new generation anticoagulants have reduced hemorrhagic effects, target-based drug design would be preferable to selectively inhibit the pathogenic effects of coagulation in the CNS. Indeed, pharmacologic inhibition of fibrin interactions with CD11b/CD18 using a fibrin peptide suppressed EAE pathology without adverse effects in blood clotting (Adams et al., 2007; Davalos and Akassoglou, 2012). Future studies will determine whether pharmacologic reagents can be developed to selectively target the pathogenic effects of fibrin and perhaps other components of the coagulation cascade in the CNS without affecting their beneficial effects in blood clotting.

## Conflict of Interest Statement

The authors declare that the research was conducted in the absence of any commercial or financial relationships that could be construed as a potential conflict of interest.

## Abbreviations

Akt: Protein kinase B
APC: Antigen-presenting cells
CCL2: Chemokine (C-C motif) ligand 2
CSPG: Chondroitin sulfate proteoglycan
CXCL10: C-X-C motif chemokine 10
EAE: Experimental autoimmune encephalomyelitis
EGFR: Epidermal growth factor receptor
ERK1/2: Extracellular signal-regulated kinase 1/2
FIE: Fibrinogen-induced encephalomyelitis
ICAM-1: Intercellular adhesion molecule 1
MCP-1: Monocyte chemoattractant protein 1
MEK: Mitogen-activated protein kinase kinase 1
NF-κB: Nuclear factor “kappa-light-chain-enhancer” of activated B-cells
PI3K: Phosphoinositide 3-kinase
ROS: Reactive oxygen species
Smad2: SMAD family member 2
TCR: T-cell receptor
TGFβ: Transforming growth factor beta
TJ: Tight junction
TLR4: Toll-like receptor 4
VCAM-1: Vascular cell adhesion molecule-1
VE: Vascular endothelial.
